# An Alternative Approach Using Nano-garlic Emulsion and its Synergy with Antibiotics for Controlling Biofilm-Producing Multidrug-Resistant *Salmonella* in Chicken

**DOI:** 10.1007/s12088-023-01124-2

**Published:** 2023-11-17

**Authors:** Azza S. El-Demerdash, Rania M. Orady, Ahmed A. Matter, Amera F. Ebrahem

**Affiliations:** 1 Laboratory of Biotechnology, Department of Microbiology, Agriculture Research Center (ARC), Animal Health Research Institute (AHRI), Zagazig, 44516 Egypt; 2 Reference Laboratory for Veterinary Quality Control on Poultry Production, Agricultural Research Center, Animal Health Research Institute, Gamasa, Egypt

**Keywords:** Nano garlic, Curli fimbriae production, Synergy, Multidrug-resistant *Salmonella*, Biofilm

## Abstract

Surface-growing antibiotic-resistant pathogenic *Salmonella *is emerging as a global health challenge due to its high economic loss in the poultry industry. Their pathogenesis, increasing antimicrobial resistance, and biofilm formation make them challenging to treat with traditional therapy. The identification of antimicrobial herbal ingredients may provide valuable solutions to solve this problem. Therefore, our aim is to evaluate the potency of nano garlic as the  alternative of choice against multidrug-resistant (MDR) *Salmonella *isolates using disc diffusion and microdilution assays. Then, checkerboard titration in trays was applied, and FIC was measured to identify the type of interaction between the two antimicrobials. A disc diffusion assay revealed that neomycin was the drug of choice. The range of nano garlic MIC was 12.5–25 μg/ml, while the neomycin MIC range was 32–64 μg/ml. The FIC index established a synergistic association between the two tested drugs in 85% of isolates. An experimental model was used including nano garlic and neomycin alone and in combination against *Salmonella* infection. The combination therapy significantly improved body productivity and inhibited biofilm formation by more than 50% down regulating the *CsgBAD, motB*, and* sipA* operons, which are responsible for curli fimbriae production and biofilm formation in *Salmonella* serotypes.

## Introduction

*Salmonella* infection is one of the most crucial and widespread infections in the world, posing a threat to the poultry industry and public health [1]. *Salmonella* is a facultative intracellular pathogen that can cause systemic or localized infections with profound negative effects on the economy and public health. The primary mode of transmission is through poultry making it the most dangerous infection for food safety in the world [2]. Many factors influence the severity of *Salmonella* infection, including host age, host immunity, presence of coinfections, infective dose, managerial issues, environmental stress, and bird age [3–5].

Antibiotics have long been regarded as the primary tools for controlling *Salmonella*, helping farmers to improve the health and growth of their livestock. However, bacterial pathogens have created and disseminated a variety of antibiotic resistance mechanisms that can spread among microbial populations questioning the use of antibiotics [2, 6, 7]. As a result, Salmonellosis therapy has become more challenging due to antibiotic resistance, that has been linked to bacterial biofilm synthesis [8–10]. Biofilm is typically defined as a structured community of bacterial cells that is adhered to an inert or living surface and encased in a self-produced polymeric matrix [11]. In reality, the adhering sessile cells within a biofilm are strongly related to host colonization, curli fimbriae production, and pathogenicity and are highly resistant to antimicrobials and host defenses [12]. Therefore, considering biofilm as a target in drug development aimed at limiting bacterial biofilm synthesis has been popular topic in the study of antibacterial infections.

In recent decades, there has been a surge of interest in employing natural products and plant chemicals as antibacterial vehicles [7, 13, 14]. Garlic tissues release an active principle called allicin, which has antibacterial activity primarily by partially inhibiting DNA and protein synthesis. This subsequently results in the complete suppression of RNA production as a major effect, and its analogs may have the potential to be further developed as gyrase inhibitors, either alone or in collaboration with other therapies [15]. Some researchers have focused on antibiofilm action and inhibitory mechanism of garlic, especially when converted to nanoparticles and combined with certain antibiotics on *Salmonella*. However, our combination could represent a new antibiofilm approach and provide insight into the molecular mechanism of drug resistance offering a great benefit to integrative medicine.

Therefore, this study was designed to investigate the synergistic effect of nano garlic combined with a drug of choice on *Salmonella* by providing a molecular extra theoretical foundation and verifying its clinical importance in limiting biofilm production and clarifying the resistance phenotype of *Salmonella*.

## Materials and Methods

### Ethics Approval

The study was designed with the permission of the Regulations of an Institutional Animal Care and Use Committee of the Faculty of Veterinary Medicine (ZUIACUC/2/F/295/2022), Zagazig University, Egypt.

### Sampling

A total of 200 broiler chickens (at slaughter age) were collected from 50 poultry farms in Damietta province that were suspected of being infected with *Salmonella* from December 2021 to May 2022. The samples were collected under strict hygienic conditions and sent to the Reference Laboratory for Veterinary Quality Control on Poultry Production (RLQP, Gamasa Lab.) as soon as possible. Internal organs (cecum, liver, and spleen) from each bird were pooled together as one sample.

### Salmonella Isolation and Identification

*Salmonella* was isolated and identified according to ISO 6579-1: 2017/Amd 1: 2020 [16]. The isolates that had been routinely recognized biochemically underwent serological identification following the Kauffman-White approach to identify somatic (O) and flagellar (H) antigens. Only smooth isolates were serologically examined and rough autoagglutinable isolates were discarded.

### Synthesis of Nano Garlic

The garlic oil (10%, Sigma-Aldrich, Cat. No. W250320) and tween 80 (surfactant) were mixed homogenously using a blender (1000 watts) for 10 min at 25 °C. Distilled water (79.4%) was then added slowly to the mixed oil phase to achieve a garlic oil micro-emulsion concentration of 20% oil in water. Nano garlic emulsion was then carried out in Nanomaterial’s Research and Synthesis Unit, Animal Health Research Institute, Dokki, Egypt [17].

### Characterization of Garlic Oil Nanoemulsion

A garlic nano emulsion was mounted on a carbon-coated grid, air dried and observed and photographed with a transmission electron microscope (TEM, JEM-2100F electron microscope, JEOL Ltd., Tokyo, Japan).

Additionally, a Zetasizer Malvern Instrument was used to test the electrical conductivity, surface charge in mV (Zeta potential), droplet size, and size distribution (polydispersity index, or PDI) of the nanoemulsion material.

### Cell Culture

Vero (or green monkey) cells were maintained in DMEM media supplemented with 10% heat-inactivated fetal bovine serum, 100 mg/mL streptomycin, 100 units/mL penicillin, and 5% (v/v) CO_2_ humidity at 37 °C.

### Cytotoxicity Assay

The Sulforhodamine B Assay (SRB) was utilized to assess cell viability with concentrations of 0.01, 0.1, 1, 10, and 100 μg/ml [18].

### Antimicrobial Susceptibility Testing

- Antibacterial Activity of Nano Garlic

Agar well diffusion assay was used to determine the effect of nano- emulsion [19]. The Muller-Hinton agar plates were inoculated with a bacterial suspension of turbidity 0.5 MacFarland standard, and holes with a diameter of 6 mm on the surface of the agar were filled with 50 µl nano garlic and incubated at 37 °C for 24 h. The inhibition zones observed were measured in millimeters. The inhibition zones of diameter less than 12 mm were recognized as having no antibacterial activity [20]

b. Disc Diffusion Method

The Kirby-Bauer disc diffusion assay was utilized to determine the antimicrobial susceptibility pattern of isolates. The confirmed *Salmonella* isolates were tested against ten commonly used antimicrobial agents in Egyptian broiler farms [21]. All *Salmonella* isolates were validated towards 11 antimicrobial drugs (OXOID) of 5 classes with the following concentrations (in μg/disk): aminoglycosides (streptomycin S, 10µg; kanamycin K, 30 µg; amikacin AK, 30µg, neomycin N, 30 µg), penicillins (amoxicillin AMX, 10 µg; ampicillin/sulbactam SAM, 20 µg), tetracyclines (oxytetracycline OT, 30 µg; doxycycline DO, 30 µg), macrolides (erythromycin E, 15µg; azithromycin AZM, 15 µg) and sulfonamides (sulfamethoxazole-trimethoprim SXT, 25µg). Susceptibility and resistance phenotypes were recognized according to the Clinical Laboratory Standards Institute's interpretation criteria [22].

c. The Determination of Minimum Inhibitory Concentration (MIC)

The MICs of nano garlic and a selected antibiotic as conducted using the microdilution broth method as described the CLSI [22]. Standard laboratory powders of drug of choice (Fisher, Bioreagents) (molecular weight, 712.722 g/mol) diluted two folds to obtain concentration of 0.125–1024 μg/ml (w/v). Nano garlic concentrations of 1.56–100 μg/ml (w/v) were also used. The MIC was determined as the lowest concentration of test material that completely inhibited microbial growth.

d. Checkerboard Method for the Combination

Serial dilutions of selected antibiotics (at least four-fold superior to the MIC) were instantly mixed in a 96-well plate containing varying concentrations of nano garlic. Additionally, 100 μl of the bacterial inoculum of 5 × 10^5^ CFU/ml then the plates were incubated at 35 °C for 24 h. Then the turbidity of each well was then noted.

The characteristics of the antibiotic interactions were determined by the fractional inhibitory concentration (FIC) indices. The FIC of each agent was detected by dividing the MIC of the drug alone by the MIC of the drug in combination. The sum of both FICs (ƩFIC = FIC of _nano garlic_ + FIC of _antibiotic_) in each well was used to categorize the combined activity of antimicrobial agents at the given concentrations as synergistic (i.e. it increases the inhibitory activity of one or both compounds in comparison with the compounds alone) (ƩFIC <  = 0.5), indifferent (i.e. there is no increase in inhibitory activity or a slight increase in inhibitory activity from the additive effect of both compounds combined (ƩFIC = 0.5–4), and antagonistic (i.e. it increases the MIC or lowers the activity of the compounds (ƩFIC > 4) [23].

### Experimental Birds

In separate cages with a biosecurity level two (BSL-2), a total of fifty healthy day-old commercial broiler chicks (Cobb) were arbitrarily distributed into five equal groups (ten chicks each). On day zero, using cloacal swabs, all groups were tested for *Salmonella* and were confirmed to be free of it. The experimental design was reviewed and approved by The Faculty of Veterinary medicine protocol in Zagazig, Egypt.

### Experimental Model Design

#### *S*almonella Challenge

All groups except the negative control were infected orally with a single dose (0.3 ml containing 8 × 10^4^ CFU) of a multidrug-resistant strain (ATCC 13311). On the fourth day, infection was performed orally by application of the same single dose per bird [24]

Chicks were assigned to five groups: Group 1: C − ve (not challenged and not supplemented with nano garlic nor antibiotic); Group 2: C + ve (challenged with S.T and not supplemented with nano garlic nor antibiotic); Group 3: G (challenged with S.T then supplemented with nano garlic at a dose of 100 mg/kg diet at 7th day for 5 successive days); Group 4: supplemented with antibiotic (recommended dose) at the 7th day for 5 successive days after being challenged with ST; and Group 5: challenged with ST then supplemented with a combination of nano garlic and antibiotic at 7th day for five successive days.

Dead chicks, clinical signs, and PM lesions were recorded daily to the end of the experiment until the 12th day of age. Productive parameters were observed according to Elsagheer [25].

Euthanasia for birds was performed using a gaseous concentration of 45% carbon dioxide to gently render them unconscious [26]. Then, the cecum was collected from each chick aseptically for *Salmonella* isolation, and enumeration according to ISO 6579-1: 2017/Amd 1: 2020.

### Quantitative Real-Time PCR Assay

qRT-PCR was performed with *Salmonella* isolates obtained from each group (Control positive and treated groups) in the Biotechnology Unit, Animal Health Research Institute, Zagazig Branch, Egypt. RNA extraction was carried out using QIAamp RNeasy Minikit (Qiagen GmbH, Germany) according to the manufacturer’s instructions. Real-time PCR amplification reaction mixtures were prepared in a final volume of 20 µL containing10 µL of 2 × Hera SYBR Green RT-qPCR Master Mix (Willow Fort, UK), 1 µL of RT enzyme mix (20), 0.5 µL of each primer of 20 pmol concentration, 3 µL of RNase- and DNase-free water, and 5 µL of RNA template. The primer sequences used for the genes involved in adhesion and biofilm formation are shown in Table 1.

**Table 1:** Primers sequences, target gene, and cycling conditions for SYBR green rt-PCR

Target gene	Primers sequences	Reverse transcription	Primary denaturation	Amplification (40 cycles)			References
				Secondary denaturation	Annealing (optics on)	Extension	
*Salmonella 16S rRNA*	CAGAAGAAGCACCGGCTAACTC	50 °C 30 min	94 °C 15 min	94 °C 15 s	60 °C 30 s	72 °C 30 s	[74]
	GCGCTTTACGCCCAGTAATT						
*csgA*	ATGCCCGTAAATCTGAAACG						[69]
	ACCAACCTGACGCACCATTA						
*csgB*	CGCATGTCGCTAACAAGGTA						[69]
	ATTATCCGTGCCGACTTGAC						
*csgD*	GATGGAAGCGGATAAGAAGC						[69]
	GACTCGGTGCTGTTGTAGC						
*motB*	AGTGGAAAAGCAGCCGAATA						[69]
	GCAACCCCTCCTGAACTAAA						
*sipA*	AGACCGAGATCAAAACGCAGG						[75]
	TCAGCGCGGGAAAATCTTC						

### Statistical Analysis

Results were expressed as mean ± standard deviation and the data were analyzed using One-way ANOVA with GraphPad Prism for windows, http://www.graphpad.com. Differences between means were detected by Tukey’s test (*p* < 0.05).

## Results

### The Recovery Rate of Salmonella Isolates from the Examined Birds

The isolation rate of *Salmonella* spp. was 10% (20/200). The highest percentage was for *S*. *typhimurium* (40%), followed by *S. infantis* and *S*. *kentucky* which gave isolation rates of 30% and 25%, respectively. *S*. *molade* was identified with a percentage of 5%.

### The Susceptibility Pattern of Salmonella Isolates to Different Antibiotics

**Fig. 1:**
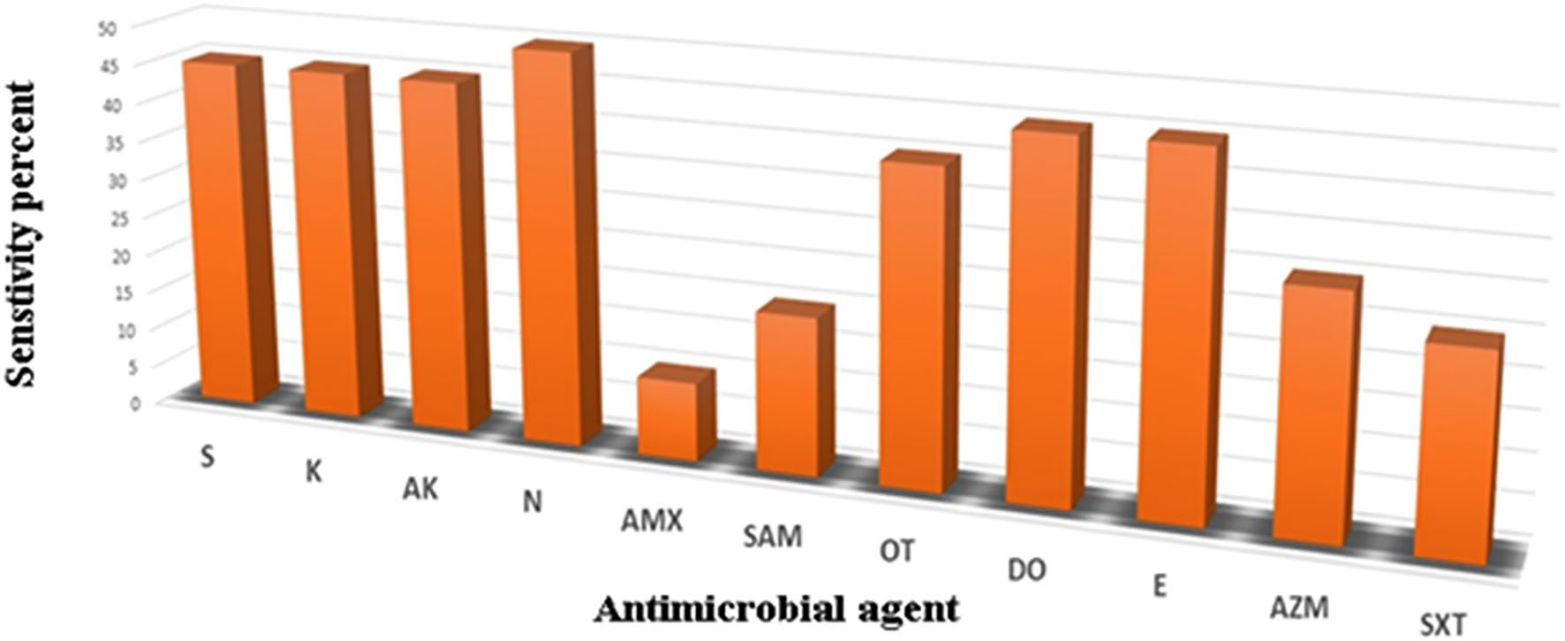
Percentage (%) of antimicrobial susceptibility of *Salmonella* isolates in the present study, *S* Streptomycin, *K* Kanamycin, *AK* Amikacin, *N* Neomycin, *AMX* Amoxicillin, *SAM* Ampicillin/sulbactam, *OT* Oxytetracycline, *Do* Doxycycline, *E* Erythromycin, *AZM* Azithromycin, *SXT* Sulfamethoxazole-trimethoprim

*Salmonella* isolates showed moderate susceptibility towards all understudy drugs as shown in Fig. 1. Moreover, all isolates (100%) were phenotypically resistant to at least three antimicrobial classes (multidrug-resistant). Neomycin represented the most profound drug among all tested antibiotics.

### Characterization of Garlic Nano-emulsion

**Fig. 2:**
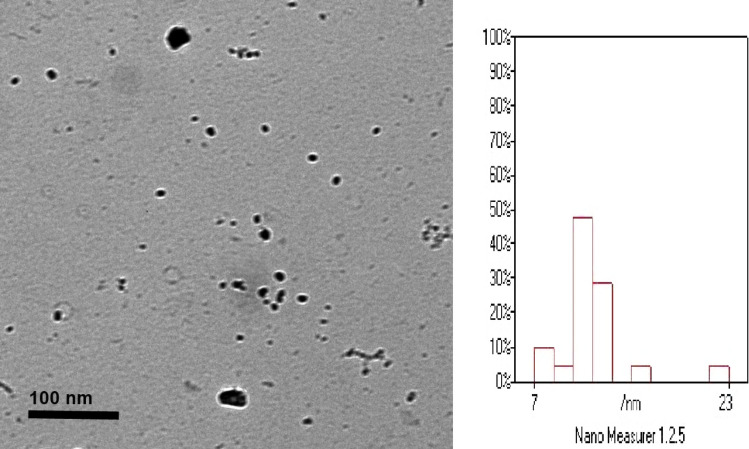
Transmitted Electron Microscopy (TEM) image showing the morphological appearance of nanoparticles dispersed. The image indicates that the particles tend to adopt a shape close to circular, exhibiting uniformity and approximate dimensional similarity

**Fig. 3:**
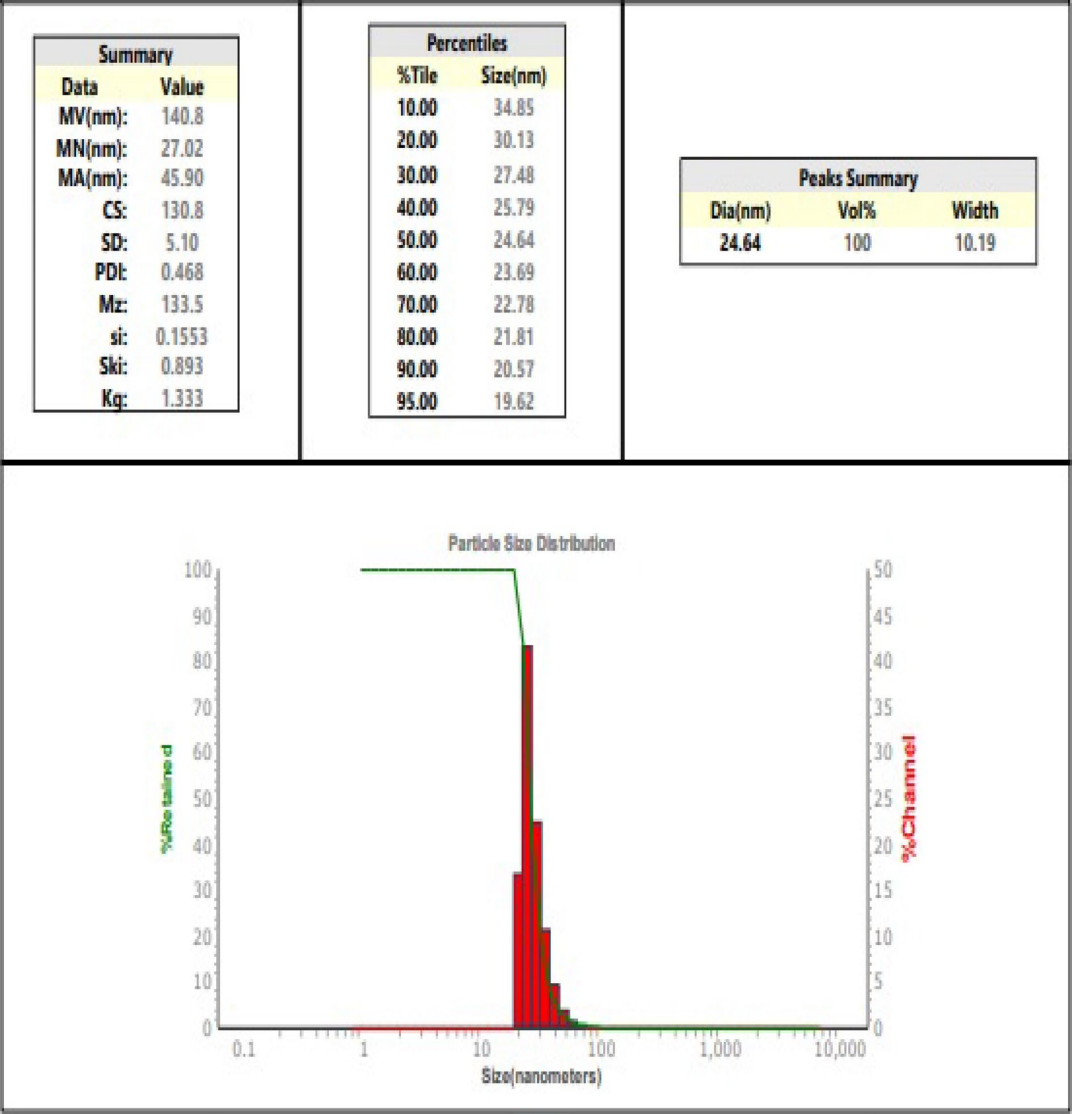
Particle size analysis of fabricate nanoemulsion of garlic using Zeta sizer Malvern Instrument

Garlic oil nanoemulsion was mainly characterized by TEM non-emulsion size, 40.94 nm with a narrow size distribution (polydispersity index: 0.165), indicating greater homogeneity in nano droplet size (Fig. 2). The zeta potential generally taken by using dynamic light scattering (DLS), indicated stable suspension, with a 7.9 mV, the same viscosity of 0.878 (cp), and conductivity of 117 uS/cm (Fig. 3).

### The Antimicrobial Potency of Nano Garlic Extract

It was evaluated by the agar-well diffusion method. It was noted that *Salmonella *spp. revealed high sensitivity against nano garlic of concentration 100% (v/v) with an inhibition zone of 17–20 mm. The microdilution assay revealed that the range of nano garlic MIC was 12.5–25 mg/mL, while the neomycin MIC range was 32–64 µg/mL as depicted in Table 2.

**Table 2:** MIC of nano-garlic emulsion and neomycin alone and in combination and FIC index against *Salmonella* isolates by the checkerboard method

Isolates no	Serotype	MIC of nano garlic	MIC of neomycin	MIC of nano garlic in combination	MIC of neomycin in combination	FIC of garlic	FIC of neomycin	Ʃ FIC	Interpretation
1	*S. typhimurium*	25	64	6.25	8	0.25	0.125	0.325	Synergistic
2	*S. typhimurium*	25	64	12.5	32	0.5	0.5	1.0	Indifferent
3	*S. typhimurium*	12.5	64	3.125	8	0.25	0.125	0.325	Synergistic
4	*S. typhimurium*	12.5	32	3.125	2	0.25	0.06	0.31	Synergistic
5	*S. typhimurium*	12.5	32	3.125	4	0.25	0.125	0.325	Synergistic
6	*S. typhimurium*	12.5	64	3.125	8	0.25	0.125	0.325	Synergistic
7	*S. typhimurium*	12.5	64	12.5	32	1.0	0. 5	1.5	Indifferent
8	*S. typhimurium*	25	32	6. 25	4	0.25	0.125	0.325	Synergistic
9	*S. infantis*	25	32	6. 25	4	0.25	0.125	0.325	Synergistic
10	*S. infantis*	25	32	6. 25	2	0.25	0.06	0.31	Synergistic
11	*S. infantis*	12.5	64	3.125	8	0.25	0.125	0.325	Synergistic
12	*S. infantis*	12.5	64	3.125	16	0.25	0.25	0.5	Synergistic
13	*S. infantis*	12.5	64	3.125	8	0.25	0.125	0.325	Synergistic
14	*S. infantis*	25	32	6.25	4	0.25	0.125	0.325	Synergistic
15	*S*. *kentucky*	25	32	6.25	2	0.25	0.06	0.31	Synergistic
16	*S*. *kentucky*	12.5	64	6.25	32	0.5	0. 5	1.0	Indifferent
17	*S*. *kentucky*	25	64	6.25	8	0.25	0.125	0.325	Synergistic
18	*S*. *kentucky*	12.5	32	3.125	4	0.25	0.125	0.325	Synergistic
19	*S*. *kentucky*	12.5	32	3.125	2	0.25	0.06	0.31	Synergistic
20	*S*. *molade*	12.5	32	3.125	4	0.25	0.125	0.325	Synergistic

### The Synergistic Effects of the Garlic Extract with Neomycin

They were determined by the checkerboard approach. It was noted that no growth or turbidity was clearly illustrated, indicating the extensive activity of nano garlic which was implemented by the second antimicrobial agent; neomycin resulting in an antibacterial effect. The synergistic behaviors of the antimicrobial combinations are listed in Table 2. The combination of nano garlic and neomycin exerted synergetic consequences against almost all isolates (n = 17) with only three being indifferent. FIC index values ranged from 0.31 to 1.5.

### Evaluation of Nano Garlic in Experimentally Infected Chicks and its Transcriptional Modulatory Effect Alone and in Combination

The entire mortality rate recorded on the 7th day post-infection within 10% of birds in groups 3 and 4 for each during the experimental period was lower than that of group 2 (30%). No mortality rates were observed in G1 (C − ve) and G5 (G + N). The clinical signs appeared on the fifth day after infection, and group 2 (C + ve) only suffered from weakness, loss of appetite, poor growth, crowding close to heat sources, drooping wings, and watery diarrhea. The post-mortem lesions were less severe in the nano garlic, neomycin, and combination-treated infected chicks as compared with the positive control infected chicks.

**Fig. 4:**
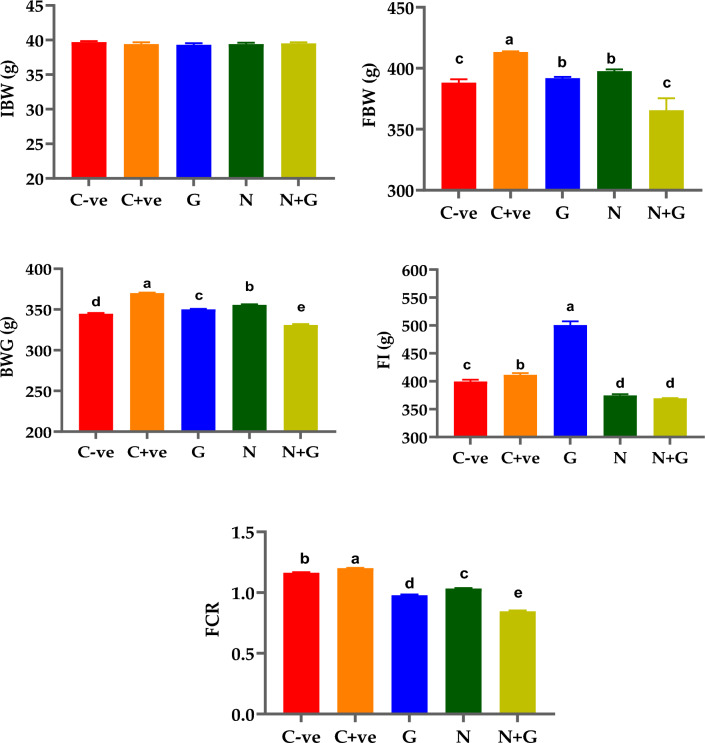
The effect of dietary garlic and neomycin in some productive parameters of broilers (12th day); Mean ∓ Standard Error (SEM) with reference to IBV/FBW/BWG/FI; *IBW* Body weight by gram at one day of age (Initial body weight); *FBW* Body weight by gram at the end of the experiment (12th day of age), *BWG* Body weight gain by gram from one day to 12 days of age; *FI* Feed intake by gram from 1 to 12 days of age, and *FCR* Feed conversion rate at 12 days of age. Different letters (a, b, c, d, e) indicate significant changes

The effects of different treatments (G, N, and G + N) on the main productive parameters were investigated in Fig. 4.

Concerning final body weight, G2 (C + ve) was significantly higher than G1 (C − ve) and the other treated group (*p* < 0.05). No significant differences were observed between G3 (G) and G4 (N). Similarly, between G1 and G5 (*p* > 0.05). For body weight gain, it was maximized in G2 (C + ve) and minimized in G5 (G + N), with significant differences between the treated group and the other unchallenged ones (*p* < 0.05). Additionally, feed utilization was affected significantly by the treatments. Feed intake was significantly higher in G2 (C + ve) than in G1(C − ve) and the other treated groups (*p* < 0.05) and no significant differences were detected between G4 and G5. The feed conversion ratio was improved significantly in G3 (*p* < 0.05) compared to the other treated and untreated groups.

*Salmonella* load reached 8.3 × 10^6^ CFU in group 2 (C + ve). This count significantly diminished in treated groups especially groups 4 and 5 as the *Salmonella* load was estimated to be less than 1 × 10^1^ CFU.

**Fig. 5:**
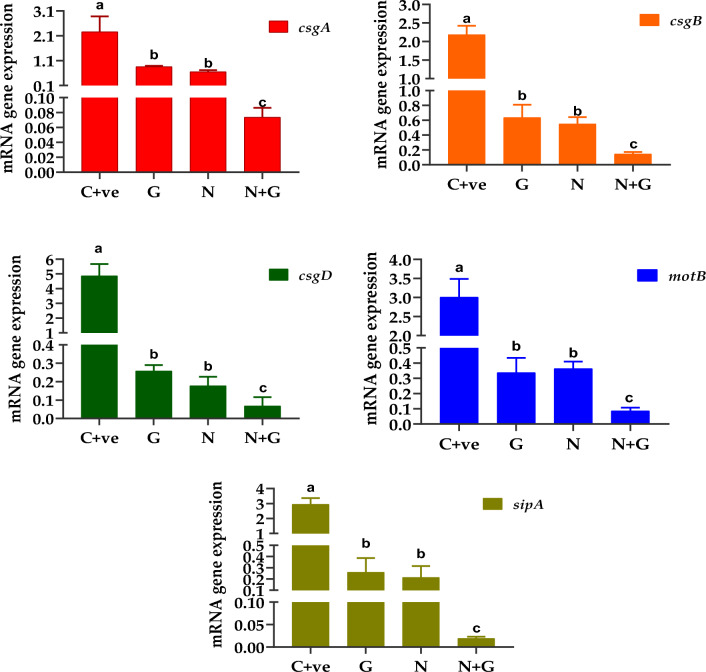
The relative mRNA expression levels of involved genes related to curli fimbriae and biofilm production through tested *Salmonella* isolates before and after treatments. Different letters (a, b, c) indicate significant changes

The quantitive expression of all studied genes was significantly (*p* < 0.05) higher in G2 (C + ve) than in treated groups. In contrast; G5 (G + N) showed significantly lower expression of all studied genes than the controls and the other treated groups (*p* < 0.05). No significant differences were detected between G3 (G) and G4 (N) (*p* > 0.05; Fig. 5A–E).

## Discussion

Salmonellosis is a zoonotic bacterial disease with national and international impacts [27]. In the current study, the prevalence rate was 10% which is consistent with previous studies by Ammar et al. [28] and Ibrahim et al. [29] in Egypt. However, a relatively higher isolation rate of 35.9% was observed in Ethiopia by Berihun et al. [30] and 37.5% in Bangladesh [31]. On the other hand, Helmy et al. [32] recorded a lower percentage of 3.4% from apparently healthy chickens in Egypt. Al-Abadi and Al-Mayah [33] recorded an overall prevalence of *Salmonella* of 5.8% in Iran. These differences in overall *Salmonella* prevalence could be attributed to several factors, including environmental management systems and seasonal patterns.

*S*. *enteritidis*, *S*. *typhimurium*, and *S*. *infantis* represent targets in control programs due to their recurrent association with human cases [34]. Additionally, *S*. *kentucky* in poultry is responsible for disease outbreaks causing significant economic losses [35]. Our seroprevalence findings were similar to several reports on the detection of nontyphoidal *Salmonella* serovars from chicken samples in Egypt [28]; India [36]; Taiwan [37] and Nigeria [38]. This variation could be attributed to the different samples examined, which could also be related to different serovars.

The stability of a nano emulsion depends on the colloidal system's behaviour which characterized by the zeta potential of the nano particles [39]. When ions from the nanoparticles diffuse throughout the solution, they attract a thin layer of ions with an opposite charge, forming a double layer. The electric potential at the boundary of the double layer, the particles' zeta potential, typically ranges from + 100 mV to  − 100 mV. As nanoparticles with zeta potentials greater than + 25 mV or lower than  − 25 mV frequently display high levels of stability [40], it has been demonstrated that nano garlic oil has a value of 7.9 mV. According to Bilia et al. [41], absorption of nano-delivery systems over the mucosa was substantially correlated with positive zeta potentials, suggesting that creating garlic oil nanoemulsion with a positive zeta potential is preferable than a negative one.

Antibiotic resistance can develop as a result of excessive antibiotic usage [3, 42, 43]. In this study, the antimicrobial resistance profile among *Salmonella* isolates was nearly similar to those recorded in Egypt [6], China [44], and Bangladesh [31].

The widespread use of antimicrobials in the poultry industry, and the emergence of MDR *Salmonella* strains that can spread to humans via the food chain, are global public health concerns [45, 46]. In this study, all isolates (100%) showed multidrug resistance phenotypes to at least three classes of antimicrobials. This percentage follows that found in Turkey [47], Spain [48], and Nepal [49]and is higher than that reported in South Africa (3.8%) [50], Iran (23.5%) [51] and Morocco (75.43%) [52].

Allicin in garlic extract has a number of advantages over most antibiotics, including the fact that it does not specifically target a protein in the bacterial cell, thus preventing the development of resistance linked to alteration of the target site [53, 54]. The antimicrobial activity of nano garlic demonstrated in this study using the agar diffusion method is consistent with previous findings [55, 56].

In the current study, nano garlic concentrations exhibited MICs of 12.5–25 μg/ml. Several studies have previously demonstrated the antibacterial potency of nano garlic against gram-negative bacteria with similar values such as Ćirković et al. [57] in Serbia, Zain al-abdeen et al. [58] in Iraq, and Amala et al. [59] in Nigeria.

We noted that the MIC of neomycin ranged from 32 to 64 μg/ml. Other records of 8 μg/ml and 32 μg/ml were previously reported [60, 61].

Combination therapies involving antibiotics and antimicrobial plant extracts offer an alternative treatment strategy for infectious diseases [62]. Nano garlic combined with neomycin reduced neomycin resistance by decreasing the MIC value, indicating a positive relationship between the two tested antimicrobial agents. However, little research has been conducted on the antimicrobial properties of the nano garlic/neomycin combination. Other studies noted strong evidence of synergism between garlic extract with ciprofloxacin [58] and vancomycin [20]. It is possible that garlic and antibiotics both inhibit similar or related sites of action in the bacterial cell [54].

The mortality rate in experimentally *S. typhimurium* infected broiler chicks was decreased after treatment with nano garlic (100 mg/mL). Postmortem lesions were less severe in the nano garlic-treated infected chicks compared with other groups. Additionally, nano garlic food supplementation significantly diminished these rates as recorded by Siddiqui et al. [63].

The *S. typhimurium* counts recovered from the cecal samples of the positive control birds ranged from 7 × 10^6^ to 9 × 10^6^ CFU of cecal contents which considerably diminished between treated groups.

This inhibition was related to the usage of garlic as dietary supplement which regulated the innate immune response to multidrug resistant *Salmonella* via several mechanisms including phagocytosis augmentation, bactericidal activity, and cytokine activation [64].

Body weight (gm) revealed a significant difference (*p* ≤ 0.05) among treated groups in comparison to the positive control group and a nonsignificant difference (*p* > 0.05) in the medians of the weight gain (gm/gm) and FCR (gm). Furthermore, a nonsignificant increase in feed intake and FCR was observed in garlic-supplemented broiler groups (3 and 5) during their infection. The effect of nano garlic feed on chicken broiler productivity was similarly documented by Ibrahim et al. [65] and Amiri et al. [55]. Additionally, Brzóska et al. [66] noted that nano garlic (2.25 mL/kg of feed) stimulated chickens' appetites, resulting in significantly higher feed intake and, as a result, higher body weight gain. Moreover, Karangiya et al. [67] indicated that garlic supplementation (10 g/kg feed) increased the absorptive surface area of the intestine and correlated with higher body weight gain in broilers. However, when comparing group 4 (N) to group 2 (C + ve) in terms of body weight and weight gain average, group 4 had a significantly higher outcome. This could have argued against the antibiotic neomycin's effect on microbial suppression which competes for nutrient material absorption when added to the diet [67].

Intensified understanding of *Salmonella* biofilm synthesis mechanisms may result in progress in the control of salmonellosis in poultry. Curli fimbriae, which are encompassed in the accumulation of rigid surfaces and improve both adhesion and cell-to-cell interactions, are represented as key protein components of biofilms [68, 69].

The *csgA *gene is involved in curli production is controlled by *csgD*, activating the *csgBAC* operon. Additionally, the presence of several essential virulence genes such as the *sipA *gene in *Salmonella,* which encodes an outer-membrane protein of the *Salmonella* pathogenicity island-2 type III secretion system vital for host infection, has been verified. Knocking out the *sipA *gene has been shown alter the ability of bacteria to synthesize biofilms [70]. Moreover, the *motB *gene plays a role in both adhesion and interaction among cell clusters [71], indicating that virulence is closely related to the ability of bacteria to form biofilms.

Transcriptional assays revealed that nano garlic alone and in combination with neomycin was able to strongly affect certain genes. This combination dose significantly downregulated the expression of these genes, indicating that G + N may inhibit bacterial attachment in the initial stages of infection and decrease the possibility of consequent biofilm production [61, 72].

Our findings demonstrate that nano garlic increases antibiotic efficacy and re-establishes newer MICs closer to the resistance breakpoints. This suggests an additional mechanism of action of the valuable outcome of complexing sub-MIC doses of nature-identical compounds with antibiotics. In light of the global extent of antimicrobial resistance, our findings present an approach to restore and preserve antibiotic efficacy [73]. This could be beneficial not only in in the poultry sector against *Salmonella* infections but also as a significant inhibitor of biofilm formation, which is considered a profound virulence factor for predominant pathogens.

## Conclusion

The results achieved in our study imply that the anti-biofilm effect of nano garlic alone and in combination with neomycin in the poultry industry can be caused by altering the bacterial surface structures and genes responsible for attachment to the conquered surface with a significant improvement of broiler performance.

## Data Availability

All data used have been included in the manuscript.
